# Semimetallization of dielectrics in strong optical fields

**DOI:** 10.1038/srep21272

**Published:** 2016-02-18

**Authors:** Ojoon Kwon, Tim Paasch-Colberg, Vadym Apalkov, Bum-Kyu Kim, Ju-Jin Kim, Mark I. Stockman, D. Kim

**Affiliations:** 1 Department of Physics, Center for Attosecond Science and Technology, Pohang University of Science and Technology, Pohang, 37673, Republic of Korea; 2 Max Planck Center for Attosecond Science, Max Planck POSTECH/Korea Res. Init., Pohang, 37673, Republic of Korea; 3 Max-Planck-Institut für Quantenoptik, Hans-Kopfermann-Strasse 1, D-85748 Garching, Germany; 4 Center for Nano-Optics (CeNO) and Department of Physics and Astronomy, Georgia State University, Atlanta, Georgia 30303, USA; 5 Department of Physics, Chonbuk National University, Jeonju, 561-756, Republic of Korea

## Abstract

At the heart of ever growing demands for faster signal processing is ultrafast charge transport and control by electromagnetic fields in semiconductors. Intense optical fields have opened fascinating avenues for new phenomena and applications in solids. Because the period of optical fields is on the order of a femtosecond, the current switching and its control by an optical field may pave a way to petahertz optoelectronic devices. Lately, a reversible semimetallization in fused silica on a femtosecond time scale by using a few-cycle strong field (~1 V/Å) is manifested. The strong Wannier-Stark localization and Zener-type tunneling were expected to drive this ultrafast semimetallization. Wider spread of this technology demands better understanding of whether the strong field behavior is universally similar for different dielectrics. Here we employ a carrier-envelope-phase stabilized, few-cycle strong optical field to drive the semimetallization in sapphire, calcium fluoride and quartz and to compare this phenomenon and show its remarkable similarity between them. The similarity in response of these materials, despite the distinguishable differences in their physical properties, suggests the universality of the physical picture explained by the localization of Wannier-Stark states. Our results may blaze a trail to PHz-rate optoelectronics.

The interest in electron dynamics in solids in strong fields has been long standing. Bloch^1^ and Zener^2^ predicted that if the electric field is large enough, the momentum of a crystal electron reaches the edge of Brillouin zone before scattering takes place, Bragg reflection occurs, and the electron is reflected toward the opposite boundary of the Brillouin zone, leading to a charge oscillation called the Bloch oscillation^3^,^4^,^5^,^6^,^7^. Wannier^8^,^9^ showed quantum mechanically that the states of a periodic crystal in an electric field originating from a single band form an equidistant spectrum called the Wannier-Stark (WS) ladder, with the spacing equal to the Bloch oscillation frequency and the wave functions localized at a single lattice site. When the electric field is sufficiently strong, competing with atomic potential gradients (on the order of ~1V/Å), the strong WS localization^8^, strong mixing of electronic bands and Zener-Keldysh type interband tunneling is expected^10^,^11^ and recently described^12^.

Due to the recent advance of laser technology, there has been a revived interest in optoelectronic phenomena in solids in strong fields^13^,^14^. In particular, a red shift of optical absorption in ZnO has been observed, which was attributed to non-parabolic band effects due to strong field^15^. High order harmonics generation in solids has been demonstrated in THz to visible^16^, UV^3^, VUV^17^ and EUV^18^,^19^ spectral region. It was successfully shown that SiO_2_ under a strong field of ~1 V/Å underwent semimetallization^20^,^21^, which manifests itself as a substantial increase of conductivity of an insulating solid above its dark value, by more than 18 orders of magnitude. It was attributed to the Zener-type tunneling transition from the valence band to the conduction band in the strong WS localization. This semimetallization turns out to be reversible on the femtosecond or faster time scale^20^,^21^. The induction of the semimetallization and its control by optical fields is of far reaching importance since it may provide a way to the development of new signal processing devices at PHz speeds^12^. An important and fundamental question is naturally whether the semimetallization occurs in different dielectrics. The fact that certain phenomena have been observed in one material does not, by any means, guarantees that it will be observed in other materials. “Is this phenomenon universal?” This is intriguing since the universality of the semimetallization phenomenon has not been previously tested and established.

Here we show the semimetallization to be a general phenomenon for wide-bandgap insulators subjected to intense optical fields. We have investigated the semimetallization in wide-bandgap dielectric crystals such as quartz, sapphire, and calcium fluoride. They exhibit a remarkable resemblance in their responses to intense optical fields despite notable dissimilarities in their composition, structure, and electronic and optical properties.

The physical properties of a solid are determined by its constituents and structure. In particular, the response of a medium to a weak external electric field is characterized by its dielectric permittivity. In this respect, quartz, sapphire and calcium fluoride are very different from each other. In fact, quartz belongs to rhombohedral lattice system with unit cell sizes of 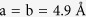, 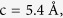 a static dielectric constant of 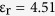, and a bandgap of 9.0 eV. Distinctively, sapphire belongs to hexagonal lattice system with unit cell sizes of 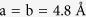, 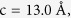 a static dielectric constant of 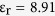, and a bandgap of 8.8 eV. Furthermore, calcium fluoride has characteristic properties different from quartz and sapphire: it belongs to cubic lattice system with a unit cell size of 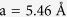, a static dielectric constant of 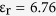, and a bandgap of 12.2 eV. In a sharp contrast to what one might have assumed from the pronounced dissimilarity of these crystals, each of them exhibits qualitatively the same response to strong ultrafast laser fields. In this work, responses of quartz, sapphire and calcium fluoride to such fields are acquired and compared under carefully-controlled conditions.

## Experiments

**Figure 1:**
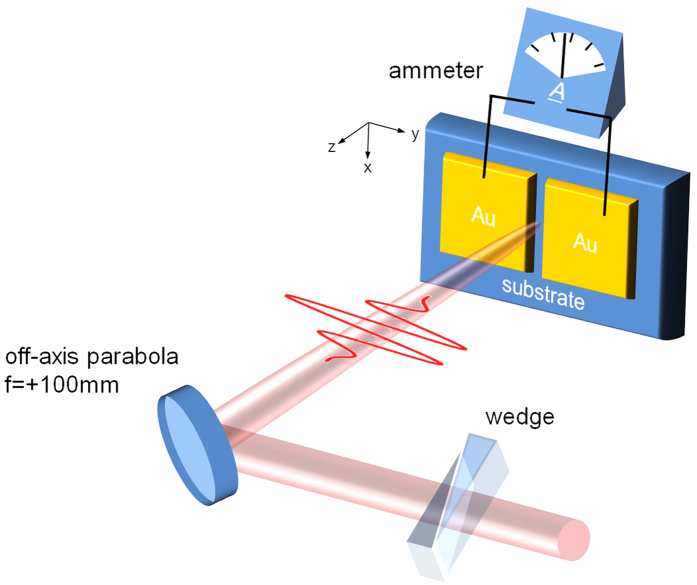
Schematic of experimental setup. CEP-stabilized few-cycle optical pulses, polarized perpendicular to the channel between the two gold electrodes, are focused onto the sample by an off-axis parabola. A wedge pair is inserted so that the optical field waveform can be adjusted by introducing an additional CEP.

Figure 1 shows the experimental schematic. Carrier-envelope phase (CEP) stable, ~4 fs optical pulses at 3 kHz repetition rate are used. The spectrum spans from 450 nm to 1000 nm, wider than an octave, and is centered at 780 nm. With an off-axis parabolic mirror, the pulse is focused onto a sample substrate, on which two 100-micron-wide metal electrodes are fabricated to face each other with a gap of 10 microns. The electrodes were made via photo lithography technique using the same chrome mask and the same recipe in order to keep them geometrically consistent. The maximum peak field strength at the focus can reach as high as ~2V/Å. Compelled by this laser field, electrons are transferred to the conduction band of the dielectric and transferred between the electrodes, resulting in a net current due to the charge transfer asymmetry. The current collected by the electrodes was amplified 10^8^ times via a current amplifier, converted into a voltage signal, and fed to a lock-in amplifier for frequency filtering. Even though attention has been paid to make the shapes of the electrodes as identical as possible, there are still slight differences microscopically between the electrodes and unequal numbers of electrons are photoemitted from the electrodes so that there is a CEP-independent charge flow. In order to remove the CEP-independent components from the transferred charge, we introduced a modulation to CEP at half repetition rate and used a lock-in amplifier referenced at the CEP modulation frequency. In this way, the CEP-independent components were eliminated and only CEP-dependent components survived. A pair of motorized wedges was included in the setup so that arbitrary CEP could be introduced. Since the comparison between materials is an important aspect in the current study, we have paid attention to deal with the purist sample. The purity is greater than 99.999% in case of quartz. As this study deals with the light intensity close to the breakdown, we were careful of laser-induced damage^22^. If a breakdown occurs during the measurement, it is manifested as a reduction in charge transfer and a scar left on the sample surface. During the measurement when the laser intensity approaches to the breakdown threshold, we have carefully checked whether breakdown arises. Whenever this irreversible damage was noticed, we abandoned the data points, in order to keep all presented data free from defects. In addition, for the better statistics, repetitive measurements were done to suppress the chance of abnormality.

### Experimental Results and Discussion

For the sake of comparison and data representation, we introduce the criticality parameter, 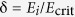, which is the ratio of internal field 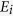 to the critical field. The response of a material to an optical field is different from material to material because the relation between the external laser field, 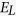, and the field inside a solid, 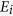, is determined by the reflection of an incident wave at the surfaces, which is self-consistently defined by the polarization inside. Hence, the field strength inside a medium, which drives electronic processes, differs from material to material at a given 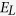 by a factor of 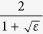. The critical field is defined as the field strength at which the potential energy change felt by an electron over a distance of the lattice spacing is equal to the band gap, 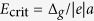, where 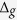 is the bandgap, 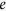 the unit charge, and 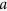 the lattice constant.

**Figure 2:**
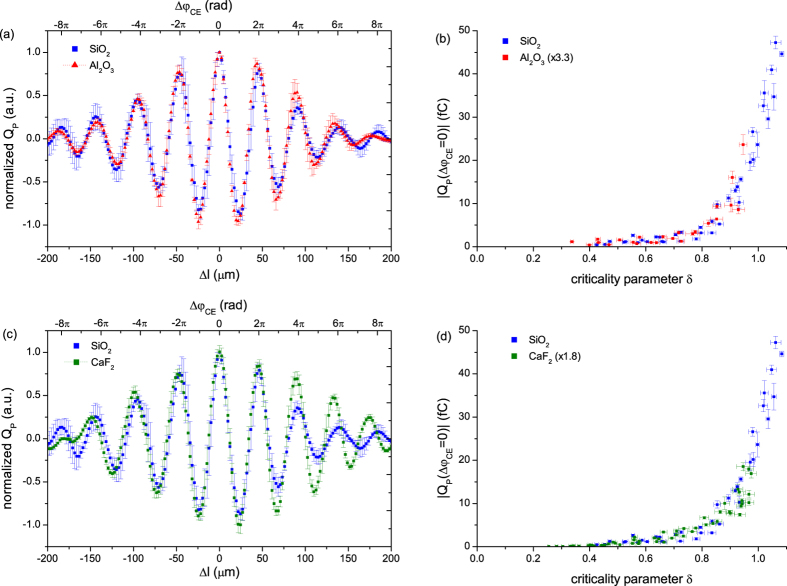
(**a**) Normalized transferred charge per pulse, 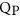, as a function of change in propagation length, 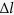, in fused silica wedge pair for an incident laser field with strength of 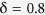 for quartz (blue) and sapphire (red). Note that 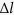 is converted into the change in CEP, 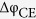, as shown in the upper horizontal axis. For 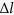 ~23 microns, the CEP is shifted by π radian^23^. Error bars show standard deviation for three independent measurements. (**b**) Measured maximum transferred charge 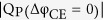 as a function of 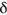. The sapphire data points are scaled by a constant factor to achieve best overlap with quartz data. Repetitive runs of measurement for different gaps on the same substrate are distinguished by different shapes of symbols. Horizontal and vertical error bars correspond to random fluctuations of laser parameters such as pulse duration, focal spot size and pulse energy and standard deviation of repetitive measurements, respectively. (**c**) and (**d**) are the same as (**a**) and (**b**), respectively, but for quartz (blue) and calcium fluoride (green).

Figure 2a shows the comparison of the CEP-dependent transferred charge per pulse, 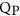, at 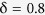 between quartz (blue) and sapphire (red). Since the their band gaps are almost the same, if there would be a difference observed, it would be due to the difference in other materials properties such as crystal structure and constituents. The CEP change, 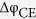, is controlled in experiments by changing the propagation length, 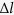, in the wedge^23^. Note that 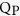 oscillates nearly periodically with 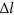. The period of these oscillations corresponds to the CEP change of 2π, implying that the measured charge transfer is sensitive to the instantaneous light field of the optical waveform. Transferred charge 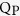 decreases as large 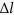 is introduced: the pulse is broadened due to the dispersion, leading to the lower peak power. As discussed for Fig. 2(b,d), 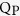 is dependent on the peak power of the driving pulse. The remarkably similar behavior of the two distinct materials in high fields strongly suggests that they are driven by the same physical processes.

The maximum transferred charge 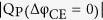 for quartz (blue) and sapphire (red) as a function of 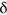 is displayed in Fig. 2b where data points for sapphire are multiplied by a constant factor, which emulates the unknown collection efficiency, to bring them to the same range as quartz’s. The horizontal and vertical error bars correspond to random fluctuations of laser parameters such as pulse duration, focal spot size and pulse energy and standard deviation of repetitive measurements for 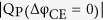, respectively. The excellent overlap of two sets of data indicates that two substances exhibit the same behavior. The multiplication factor of 3.3 implies that more charge transfer is detected in quartz than in sapphire at a given 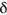. This factor results from the combined contributions of the difference in the amount of charge generated inside materials and the junction characteristics which include the electrical contact resistance between the dielectric substrate and metal electrodes, and the collection efficiency of electrodes, in particular.

Since quartz and sapphire have almost the same band gap but different lattice systems, the good agreement between quartz and sapphire indicates that the structural factors may not play a major role in this phenomenon. Another question is whether it is the different band gap that really matters. To answer this, the comparison with the data from calcium fluoride sample is made: as mentioned earlier, the calcium fluoride has a different lattice system (cubic lattice) and a larger band gap (12.2 eV). Figure 2c,d show such a comparison between quartz and calcium fluoride. We note that in Fig. 2a,c, there is more discrepancy between quartz and calcium fluoride than between quartz and sapphire at large 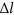. It is resulted from dispersive broadening of optical pulses. Figure 2d shows the transferred charges with respect to 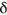 for both quartz and calcium fluoride. Similar to what we described above in conjunction with Fig. 2b, the data points for calcium fluoride are multiplied by a constant 1.8 to fit them to the data for quartz. Again, an excellent overlap of the data points for these two solids is observed implying that calcium fluoride and quartz behave essentially in the same way; all curves increase nearly exponentially with 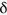.

**Figure 3:**
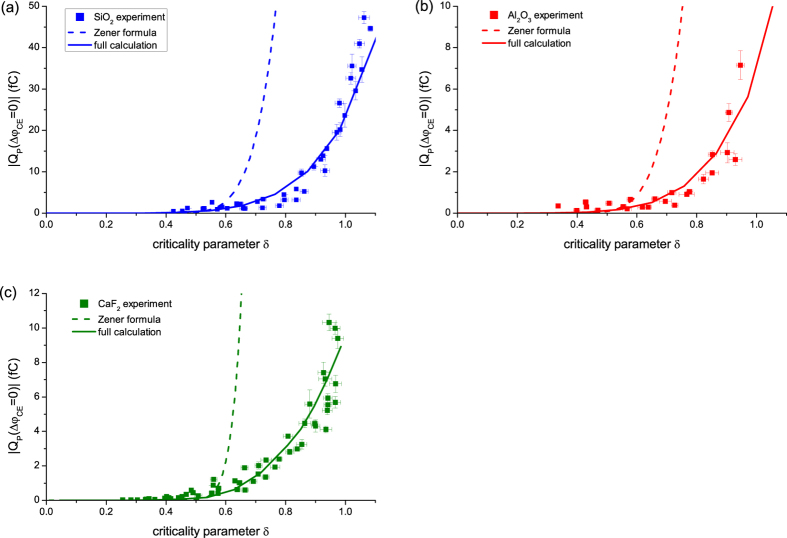
Measured maximum transferred charge 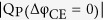 as a function of 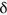 (squares); fitting based on Zener-Keldysh interband tunneling formula (dashed) and theoretical computations (described in the text) including both interband and intraband transition (solid) for (**a**) quartz, (**b**) sapphire and (**c**) calcium fluoride. Error bars represent the same information as described in the caption of Fig. 2b.

Zener has studied interband charge transfer, in terms of electrical breakdown in an insulator under strong field and derived an analytical formula^11^. Based on this, the transferred charge is simulated as 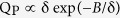, where 
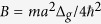
, and 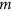 is electron mass. We have attempted to fit this formula to our experimental data. Since Zener has considered up to the 1^st^ order of Fourier expansion for periodic potential, field strength was assumed to be small. Hence we attempted to better fit a lower-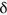 region (see more details in Method). In each panel of Fig. 3, this fitting (dashed curve) is superimposed with the measured data. It is evident that the Zener tunneling does not explain measured 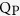 against 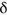; experiment and Zener’s formula are in a severe mismatch. Zener’s theory appears to overestimate the population of electrons promoted from the valence to conduction band compared to the measurement.

To theoretically describe the measured data at high fields, i.e., in the higher 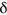 region, a series of numerical simulations was performed taking into account both interband and intraband transitions. The calculation is based on a tight-binding model for the atoms at their actual positions in the unit cell (see more details in Method). The Schrödinger equation for electrons in a strong field and Maxwell equations are solved in parallel using the finite difference time domain (FDTD) method. General details of the technique are published elsewhere^21^,^24^,^25^. The model parameters were chosen to fit the band structure, and known linear optical properties of materials under study were correctly described, and no further modification of these parameters was made. Time-dependent Schrödinger equation has been solved taking electron interaction into account only through macroscopic electric field and neglecting electron-electron collisions, which is justified by the very short, ~1 fs, time scale of the interaction and very large values of the optical field, 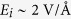, which are greater than internal fields in the crystals. In parallel, Maxwell’s equations, coupled with the Schrödinger equation through polarization and optical electric field, are solved by the FDTD method using parallel computing. From the above-described computations, we calculated current density 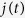 as a function of 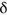. By integrating 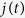 over time, we obtained the charge density 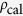.

To compare theory and experiment, 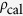 is multiplied by an effective cross section 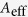 to obtain 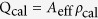, which corresponds to experimentally measured charge transfer 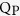. 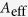 is a proportional constant to match values and dimensions of computed and measured charge, considering factors which could not be included in the calculation. The factors which influence 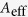 might be (1) mobility of a material, (2) property of electrical contact between dielectric substrates and metal electrodes, (3) microscopic imperfection in geometry during fabricating the electrodes or (4) misalignment of sample with respect to polarization axis or focal position.

According to the computation, the largest charge transfer is predicted for calcium fluoride at a given 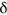: greater than in quartz by 24 times and than in sapphire by 36 times. But it does not agree with experiment. The greatest charge transfer is measured in quartz, 3.3 times larger than sapphire and 1.8 times more than calcium fluoride. 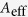 of quartz is the largest, implying that it is most probable for electrons to be captured by the electrodes and detected as current in quartz. The values of 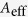 which ensures the best overlap to experimental data are 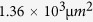 for quartz, 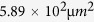 for sapphire, and 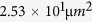 for calcium fluoride, respectively.

By multiplying proper 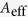, the result for each material is plotted as a solid line in Fig. 3. We notice that our calculations fit the measured data points considerably better than the Zener-type tunneling curve. The contribution of intraband transition, which was irrelevant for the case of Zener’s formalization, amounted to 85% of total charge transfer at fields of δ~0.5 and it became even more dominant, coming to about 95%, at a higher fields (δ∼1.0). We note that (i) near-exponential increase is reproduced in simulations, in accord with the experiment and (ii) the good overlap of the experimental data with calculation results can be achieved by employing constant effective cross sections for the different materials. These imply that quartz, sapphire and calcium fluoride respond to the internal optical electric fields essentially in the same manner, despite the fact that they are very different in chemical composition, physical and crystallographic properties.

Even though the microscopic description presented above provides good description of the experimental data, we would like to build a physical picture of the phenomena observed, especially to explain qualitatively why the very dissimilar dielectric crystals exhibit very similar behavior in strong fields suggesting universality of this phenomenon and underlying physics.

We interpret this universality as a result of the WS localization^9^. This is an adiabatic phenomenon occurring when electrons in a given band are accelerated to the boundary of the Brillouin zone and reflected back experiencing the Bloch oscillation^1^ at a frequency of 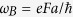. Under our conditions, for 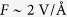 and 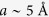, the Bloch frequency is on the order of the bandgap, 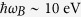. The WS states originating from a single band form an equidistant spectrum with spacing equal to 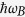 called WS ladder.

The condition of adiabaticity is that the optical frequency, 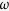, is much less than the level spacing. At low fields, it is well satisfied for wide-bandgap dielectrics. 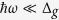. At high fields, the adiabaticity condition is 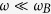, and it is also well satisfied under our conditions. Thus the adiabatic picture of the WS states is well applicable both for low and high fields.

All WS states are localized within localization radius 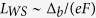, where 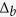 is the energy width of a band (valence or conduction). For maximum fields 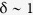, 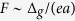, and, assuming realistically that 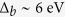, we estimate 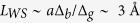. Thus, under our conditions, the WS localization is strong – within a unit cell. Such a strongly localized electron does not explore the long-range order and symmetry of the crystal. This qualitatively explains the observed universality – insensitivity of the semimetallization phenomena to the crystal structure.

## Conclusion

In conclusion, we have shown experimentally that wide-bandgap insulators, quartz, sapphire and calcium fluoride, undergo semimetallization in strong optical fields: there is charge transfer along the field direction, which is dependent on the maximum instantaneous field and is controlled by the CEP of the pulse. For these crystals, the semimetallization occurs in a very similar way, although their chemical compositions, crystallographic, and linear-optical properties are quite dissimilar. These observations can be understood in view of the WS localization of electrons. Since under our conditions, fields of 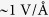, the WS localization is strong, confining the electron wavefunction within the lattice unit cell, the electrons do not explore the symmetry and long-range order; thus the semimetallization occurs in the same way regardless of the detailed structure of the crystals. The semimetallization is not so well-described by Zener-Keldysh tunneling formula and the mismatch gets even more for high fields. This is because it is indeed intraband transition which dominates but is neglected in derivation of Zener’s tunneling rate formula. The quantum mechanical simulations including both inter-and intraband transition and population dynamics do describe the semimetallization and its similarity among different crystals. This study of the semimetallization controlled by optical field potentially opens a door for electron manipulation and signal processing on the sub-femtosecond time scale using wide-bandgap dielectric materials. To understand the ultrafast semimetallization of insulators under strong field in another dimension, optical pump-THz probe measurement, which has been successful^26^,^27^,^28^, will be useful. Conductivity of an insulating solid strongly depends on frequency. Terahertz (THz) measurements provide conductivity at much lower frequencies, which, together with investigation in optical frequency region, will enhance our understanding. Also, strong THz fields by themselves can cause a multitude of nonlinear phenomena^16^. This is a separate wide field that deserves a separate study.

## Method

### Details of Theoretical Computations

We assume that the electron dynamics in external electric field of the optical pulse is coherent (which means that the duration of the laser pulse is less than the characteristic electron scattering time) and can be described by the time dependent Schrödinger equation


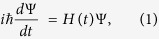


where the Hamiltonian 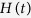 has explicit time dependence and has the following form


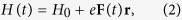


where 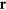 is a three-dimensional vector, 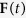 is the electric field of the optical pulse, and 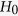 is the free-electron Hamiltonian, which is described by the tight-binding model and has the size 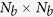. Here 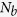 is the number of the basis orbitals per unit cell and also is the number of valence and conduction bands taking into account in our calculations. The advantage of the tight-binding model is that it captures the symmetry properties of the solid.

The free-electron Hamiltonian 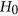 determines both the energy spectrum, 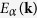, 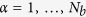, and the corresponding wave functions, 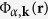, of a free electron in the solid. It is convenient to solve the time-dependent Schrödinger equation in the basis of Houston functions, which are given by the following expression


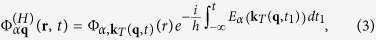


where


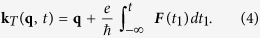


The Houston functions are solutions of time-dependent Schrödinger equation (1) within a single-band approximation. They describe the intraband electron dynamics in time dependent electric field. Using the Houston functions as the basis, we express the general solution of the Schrödinger equation (1) in the following form


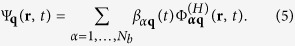


The expansion coefficients 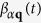 satisfy the following system of differential equations


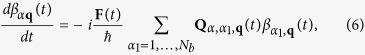


where 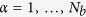, and vector-function 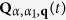 is proportional to the inter-band dipole matrix elements


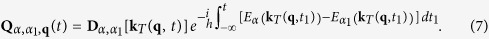


Here 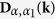 are the dipole matrix elements between the states of bands 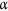 and 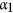,


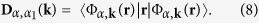


It is easier to calculate the dipole matrix elements in terms of the interband matrix elements of the velocity operator,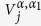. The velocity operator is defined by the following expression:


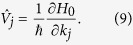


Then the dipole matrix elements are defined as


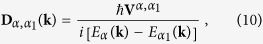


where 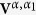 is velocity-operator matrix element.

System of equations (6) determines electron dynamics in the time-dependent field of the laser pulse. For numerical solution of the problem we consider 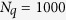 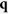-points in the reciprocal space. For each 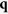
**-**point, system of equations (6) has dimension 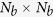. We solve 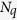 such systems numerically by fourth order Runge-Kutta method. From the solutions of systems of equations (6) we obtain reciprocal space distribution of electrons in the conduction and valence bands.

From these electron distributions we can find the current generated in the system in the field of the pulse,


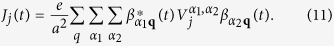


The current is determined by the matrix elements of the velocity operator. The generated current results in the charge transfer through the system, which is determined by an expression


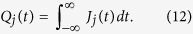


For corundum (Al_2_O_3_), the primitive unit cell has 15 atoms (three Al_2_O_3_ units). We take into account the s- and p-orbitals for each atom and use the corresponding parameters of the tight-binding Hamiltonian reported in Ref. 29. The parameters of the model in notations of Ref. 30 are shown in Table 1.

For CaF_2_, we consider the minimal tight-binding model, which includes only s and p states for Ca and F. The parameters are given in Table 2 (see Ref. 31).

The quartz (SiO_2_) is modeled in a virtual crystal approximation with the corresponding tight-binding parameters given in Table 3 (see Ref. 32).

### Fitting with Zener’s tunneling rate formula

Zener’s formula was derived based on assumptions and approximations. For example, (1) a lattice is simplified to one dimension, (2) the periodicity of potential is considered up to the 1^st^ order in Fourier expansion, and (3) the applied field is not too strong that the energy change for displaced electrons in the crystal is rather still small. The field strength in which Zener’s approximation is valid is, considering typical lattice constants, estimated to be on the order of a few volts per nanometer, or 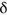 is a few tenths. The threshold value below which Zener formula is valid is not clear. We have done several fittings with data for small 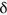.

**Figure 4:**
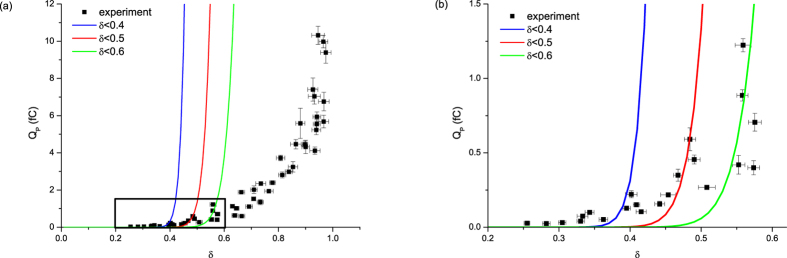
(**a**) Fitted curves by Zener’s formula for transition probability multiplied by a constant which ensures least discrepancy to the experimental data for δ smaller than a value given in the legend for calcium fluoride (**b**) magnified view for black rectangle in the left panel.

The data for small 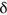 is limited because signals become so small that they are buried in noise. Yet we still have meaningful data for 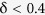 in the case of calcium fluoride. Such fittings were done for calcium fluoride. The results are shown in Fig. 4, We fit experimental data points for up to 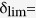 0.4(blue line), 0.5(red line) and 0.6(green line) using Zener’s formula. The fitting to the data for small 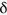 is zoomed in and shown in the right panel. The agreements are not good and all curves skyrocket near its threshold 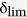. It is clear that Zener formula is not valid for the field strength corresponding to 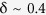, not to mention the field of 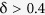. Quartz or sapphire have only a few data points for 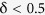 since signals are weaker. Hence we have tried to fit data for 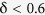 for all samples to clearly demonstrate that Zener formula cannot describe the data for high field.

## Additional Information

**How to cite this article**: Kwon, O. *et al.* Semimetallization of dielectrics in strong optical fields. *Sci. Rep.* **6**, 21272; doi: 10.1038/srep21272 (2016).

## Figures and Tables

**Table 1:** The nearest-neighbor tight-binding parameters for Al_2_O_3_ in eV.

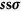	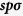	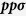	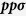	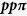
−3.117	4.097	7.214	3.776	−1.803
	**E (s)**		**E (p)**	
O	−29.14		−14.13	
Al	−10.11		−4.86	

**Table 2:** The nearest-neighbor tight-binding parameters for CaF_2_ in eV.

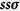	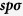	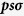	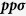	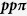
−1.139	0.36	1.086	3.776	−0.088
	**E(s)**		**E(p)**	
Ca	5.75		10.4	
F	−30.91		−8.0	

**Table 3:** The nearest-neighbor tight-binding parameters for SiO_2_ in eV.

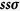	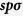	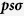	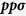	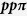
−2.27	3.927	3.927	5.466	−0.314
	**E(s)**		**E(p)**	
SiO_2_	4.8		1.83	
